# *In situ* chamber for studying battery failure using high-speed synchrotron radiography

**DOI:** 10.1107/S1600577522010244

**Published:** 2023-01-01

**Authors:** Jonas Pfaff, Matilda Fransson, Ludovic Broche, Mark Buckwell, Donal P. Finegan, Stefan Moser, Sebastian Schopferer, Siegfried Nau, Paul R. Shearing, Alexander Rack

**Affiliations:** a Fraunhofer Institute for High-Speed Dynamics, Ernst-Mach-Institut, EMI, Efringen-Kirchen, Germany; b ESRF – The European Synchrotron, Grenoble, France; c Electrochemical Innovation Laboratory, Department of Chemical Engineering, University College London, London, United Kingdom; d The Faraday Institution, Harwell Science and Innovation Campus, Didcot, United Kingdom; e National Renewable Energy Laboratory, 15013 Denver West Parkway, Golden, CO 80401, USA; Bhabha Atomic Research Centre, India

**Keywords:** X-ray imaging, Li-ion batteries, safety, thermal runaway, *in situ* studies, abuse tests, propagation

## Abstract

The investigation of lithium-ion battery failures is a major challenge for personnel and equipment due to the associated hazards (thermal reaction, toxic gases and explosions). To perform such experiments safely, a battery abuse-test chamber has been developed and installed at the microtomography beamline ID19 of the European Synchrotron Radiation Facility (ESRF). The chamber provides the capability to robustly perform *in* *situ* abuse tests through the heat-resistant and gas-tight design for flexible battery geometries and configurations, including single-cell and multi-cell assemblies. High-speed X-ray imaging can be complemented by supplementary equipment, including additional probes (voltage, pressure and temperature) and thermal imaging. Together with the test chamber, a synchronization graphical user interface was developed, which allows an initial interpretation by time-synchronous visualization of the acquired data. Enabled by this setup, new meaningful insights can be gained into the internal processes of a thermal runaway of current and future energy-storage devices such as lithium-ion cells.

## Abstract 2

The developed test chamber, capable of high-speed X-ray imaging, was designed to perform robust *in situ* abuse tests on batteries in various configurations while providing a complete overview of the failure event with additional complementary measurement methods.

## 1. Introduction

Energy storage can be considered one of the key technologies of the 21st century. The increasing number of electric vehicles and the associated demands for cost-efficient energy storage or the reduction of carbon emissions through the effective storage of energy from renewable sources are just two of the relevant challenges. Battery technology, including questions of reliability and safety, is one of the most central aspects of these challenges, which will become even more important in the future due to the ever-increasing market share of electric vehicles (IEA, 2021 ▸; Bernard *et al.*, 2021 ▸) and the large-scale application of batteries as energy storage in consumer electronics such as smartphones or laptops; the increasing demand but also the increasing power requirements for the energy-storage medium must be met. The electrochemical nature of lithium-ion batteries and the resulting high energy density of the battery cells make this technology ideally suited for use as an effective energy-storage medium (Blomgren, 2017 ▸; Bandhauer *et al.*, 2011 ▸).

However, the high energy density of Li-ion cells also contributes to major safety concerns. Although the probability of Li-ion cells undergoing thermal runaway is extremely low, there have been some reports of serious damage in the recent past (Sun *et al.*, 2020 ▸). If a worst-case scenario is considered, the flammables and oxidizers contained in the batteries can trigger an exothermic runaway reaction, known as thermal runaway (TR), and may even result in an explosion (Spotnitz & Franklin, 2003 ▸; Wang *et al.*, 2012 ▸). A TR can be initiated by the following causes: mechanical abuse, thermal abuse, incorrect charging or internal and external short circuit (Finegan *et al.*, 2017*a* ▸; Bandhauer *et al.*, 2011 ▸; Ouyang *et al.*, 2019 ▸; Doughty & Roth, 2012 ▸). Consequently, the need for investigations in a wide range of applications of these worst-case scenarios is increasing in order to better understand the internal processes during a TR and, consequently, to make improvements to the safety measures. For this reason, battery safety is the focus of several research and industry projects around the world to further develop the current state-of-the-art towards safer Li-ion batteries (Grey & Hall, 2020 ▸; Armand *et al.*, 2020 ▸).

To advance this research, it is crucial to holistically understand both the processes within a single cell and the interactions between multiple cells (propagation of TR). To elucidate these complex effects, various experiments have been conducted using different experimental setups, *e.g.* triggering by mechanical [*e.g.* nail penetration (Finegan *et al.*, 2017*b* ▸)], thermal or electrical stress (*e.g.* defined internal short circuit). The results can then be used to create accurate models of battery cells or cell assemblies (Wang *et al.*, 2022 ▸; Finegan *et al.*, 2019 ▸). In this regard, high-speed synchrotron X-ray radiography is recognized as a highly valuable tool for studying the rapid internal events during TR reactions, and is being used by research groups to better understand the characteristics (Finegan *et al.*, 2015 ▸, 2017*a* ▸; Pham *et al.*, 2020 ▸). This is due to the detailed visualization of gas accumulation, along with the structural collapse and material degradation during the TR: by using hard synchrotron radiation with (partial) spatial coherency as illumination, together with a broad bandwidth (polychromatic), not only can one achieve higher acquisition rates (and higher temporal resolution) but also higher sensitivity (leading to a better image quality) compared with laboratory-based sources. High-speed radiography allows the functionality of integrated safety mechanisms to be evaluated *in* *situ* (Finegan *et al.*, 2018 ▸).

In order to carry out such experiments safely, a suitably prepared and safe laboratory environment is required that is able to handle the expected dangers and at the same time allows precise measurements. Since the danger potential for TR scales with the energy content of the cells, the challenge and necessary infrastructure to create a safe environment increase significantly for larger cells. One possible approach to this challenge is the concept of a safe micro-environment that is robust enough to safely contain TR events, which allows observation of the experiment with different measurement techniques and which can be integrated into complex laboratory environments such as, for example, a synchrotron.

The solution presented in this paper is a robust *in* *situ* abuse-test bench suitable for high-speed X-ray imaging of fast and high-energy battery failure events The X-ray imaging will help us to improve our understanding of the spatial dynamics of TR and validate existing TR models to further improve the safety of battery modules and cells. The design is flexible enough to be adapted to new cell materials, new diagnostic methods and new safety devices to investigate batteries of current as well as emerging technologies. In addition to high-speed X-ray imaging, the test chamber allows the application of further complementary measurement techniques (such as temperature, pressure, voltage, gas analysis), enabling the user to correlate the different processes and draw comprehensive conclusions. The battery abuse-test chamber is designed to allow containment and observation to continue after the initial TR; this also allows the cell-to-cell propagation of TR to be studied, which has received very little attention so far (at least using *in situ* X-ray imaging) due to the experimental challenges associated with this process. A better understanding of the process of propagation is deemed essential for improving the safety of battery modules, since slowing down or even stopping propagation is one of the key safety features. The experiments conducted with this test chamber are intended to advance the state of research and development of next-generation battery design and help to launch new innovative technologies. The chamber is available for user experiments at the European Synchrotron ESRF (France), at beamline ID19 in the framework of the public programme.

## 2. Battery abuse-test chamber

**Figure 1:**
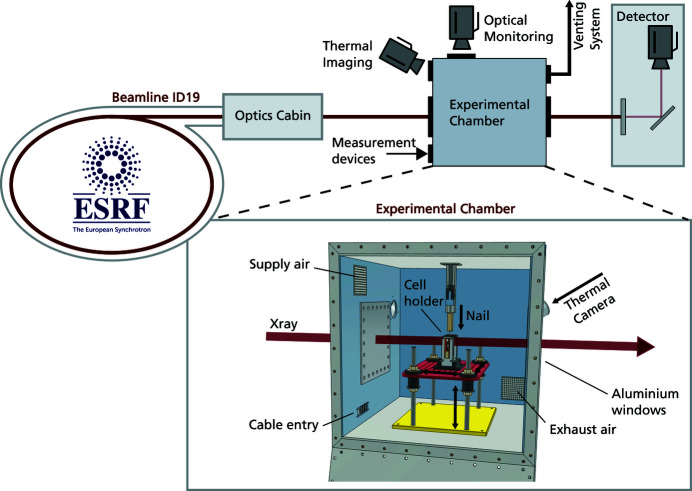
Sketch of the battery abuse-test chamber installed at the ESRF beamline ID19 including an overview of the setup at the beamline and a detailed description of the chamber.

The battery abuse-test chamber was developed to extend the battery research opportunities at the ESRF and in particular to combine battery abuse testing with high-speed X-ray imaging. An overview of the testing chamber and its integration into the existing X-ray imaging facility at ID19 at the ESRF can be seen in Fig. 1 ▸. The first adaptation includes a nail device for triggering an internal short circuit by nail penetration. In addition, further access ports for a variety of complementary measurement technologies are implemented. Furthermore, a comprehensive safety protocol has been established which allows these challenging experiments to be carried out in a routine manner.

**Figure 2:**
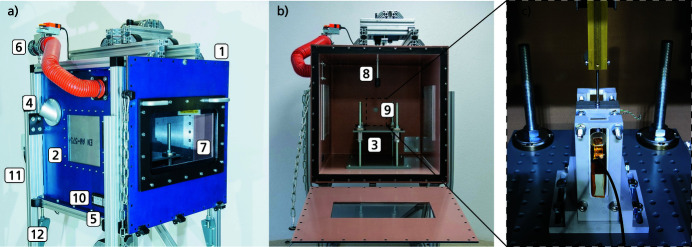
Pictures of the battery testing chamber, showing in (*a*) the outside of the chamber, with the extraction tube and valve visible, as well as the polished X-ray aluminium windows, and the opening for the thermal camera. In (*b*) the inside of the chamber with the sample table and nail piston is visible. The cell holder which is placed on top of the sample table is depicted in (*c*) with the nail going through the guiding hole and the cells mounted inside the cell holder.

Due to the aggressive nature of Li-ion battery failures, and consequently the risk involved in performing related experiments, a testing chamber and the setup around the chamber have been designed and constructed by the Fraunhofer Ernst-Mach Institute (Germany) to allow the experiments to be carried out in a safe manner. In particular, the chamber can contain fire, tolerate a significant pressure increase, ventilate toxic gases and particles, and additionally has the capacity to suppress fire by purging with inert gas. The chamber shown in Fig. 2 ▸ consists of the main testing chamber (1), equipped with two 6 mm-thick X-ray-transparent polished aluminium windows (2), a sample table (3), and two germanium windows for thermal imaging attached to the outer end of the adapters (4). The design of the chamber was chosen so that the cells vent in a direction where no sensitive instruments and surfaces are located. This reduces the stress on the aluminium window and the germanium window to such an extent that replacement will not be necessary during series of experiments. In addition, the aluminium window inside the chamber is protected with a layer of Kapton foil to prevent scratches. Furthermore, the chamber contains an automatic air/inert gas inlet (5), a controllable ventilation inlet (6), a door with a window (7), two mounts for the nail penetration system (8 and 9) and a gas-tight connection box (10). The connectors between the cables inside the chamber and the connection box were placed in the lower area under the sample table, widely protected from possible damage by the TR. The height-adjustable legs (11), placed on rails (12), both increase the testing flexibility of the chamber. The chamber has a total internal volume of 343 l (700 mm × 700 mm × 700 mm), constructed from three sidewalls of 6 mm-thick stainless steel, welded to the 8 mm-thick top and bottom panels. The 8 mm-thick front panel has a bottom-hinged door to facilitate sample changes and cleaning of the chamber. The exhaust pipe is connected to an extraction unit, equipped with filters that are suitable for filtering dust particles as well as hydrofluoric acid (HF) fumes. The inlet valve is connected to a bottle of carbon dioxide that can be automatically purged into the chamber to suppress fire. This setup is available at beamline ID19 of the ESRF, to exploit the beamline’s unique capabilities of high-speed X-ray imaging. Moreover, as previously mentioned, temperature/voltage measurements and thermal imaging are easily available in addition to radiography.

**Figure 3:**
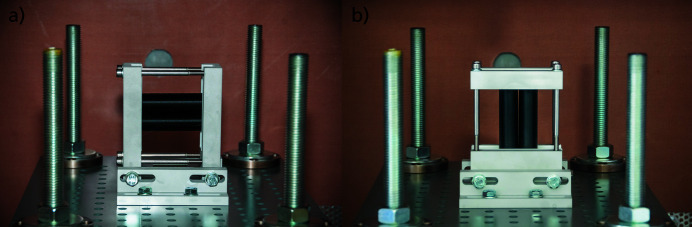
Image of the sample table including the sample holder. The dummy cells are shown in (*a*) a horizontal configuration and (*b*) a vertical configuration. The current setup includes variable positioning of the cell holder, height adjustment of the sample table and different configurable cell holders as well as two different positions for the nail devices: a flexible positioning of the initiation mechanism and high-speed X-ray radiography with cell configurations of up to four cylindrical cells.

The enclosure has been successfully tested up to a pressure increase of 800 mbar and a temperature of 800°C. With an estimate of 3 l gas production per Ah (battery-cell capacity), the chamber is suitable for testing one single cell or multiple cells. The sample table has a flexible mounting area for different sample holders, made specifically for the type of battery model or configuration to be tested. An example of a sample holder can be seen in Fig. 3 ▸, showing (*a*) a horizontal configuration and (*b*) a vertical configuration of two cylindrical cells. This cell holder consists of several parts. The basic structure is an aluminium frame, which ensures mechanical stability and robustness during the test. This frame has no direct contact with the surface of the cells. The contact is realized by inserted 3D-printed inlets. These inlets provide an electrical isolation between the fixture and the cell; furthermore, these inlets were designed in such a way as to allow fixation without affecting cell behaviour during TR. Thus, although the fixture which holds the cell on each side has only 2 mm contact area, the cell still remained in place during nail penetration.

## 3. Data acquisition

Studying battery failure by means of abuse testing with high-speed X-ray radiography requires a high photon flux in the hard X-ray regime as well as sufficient beam size (frequently 2000 frames s^−1^ up to 20000 frames s^−1^ are sufficient to capture the relevant dynamics). In addition to the rather dense batteries, the flux must be transmitted through the aluminium safety windows of the chamber, which are several millimetres thick; therefore the photon flux needs to be high enough so that sufficient photon intensity still reaches the (indirect) X-ray image detector. As spatial resolution requirements increase, so does the need for high photon flux as smaller areas of the beam are magnified onto the camera’s pixels. At beamline ID19, the installed wiggler (type w150) represents a good compromise between high photon flux density at the required hard wavelengths and a sufficiently large and homogeneous beam (Weitkamp *et al.*, 2010 ▸; Rack *et al.*, 2013 ▸). Because of the 150 m-long distance between the source and the experiment at beamline ID19, the wavefront at the position of the experiment allows the user to work with so-called propagation-based phase contrast due to its (partial) spatial coherency (Cloetens *et al.*, 1996 ▸; Espeso *et al.*, 1998 ▸). For the spatial resolutions relevant to battery abuse testing as shown here, frequently a drift space between the sample and detector of up to 8 m is applied. Hence, phase contrast by means of edge enhancement is present in the images which substantially improves the contrast without distorting geometrical information. In case less attenuating batteries are to be studied, two undulators (type u32) are available with higher photon flux density at lower photon energies which would allow the user to reach higher spatio–temporal resolution (Olbinado *et al.*, 2017 ▸).

For high-speed X-ray imaging, beamline ID19 utilizes indirect detection schemes: a scintillator screen converts X-rays into visible light; this luminescence image is projected (magnified) by visible-light lenses onto the sensor of a (digital) camera (Rack *et al.*, 2010 ▸). Depending on the desired frame rate, different cameras are available: sCMOS-based cameras such as pco.edge (PCO AG, Germany) reach up to 100 frames s^−1^, for higher acquisition rates up to several 100 kHz a SA-Z by Photron (Japan) is available and for MHz frame rates there is a HPV-X2 (Shimadzu, Japan). A broad variety of visible-light lenses can be used to couple the luminescence image of the scintillator magnified or demagnified to the camera sensor. Magnification ratios range from 1× to 20× and demagnification from 1× to 3×. The material of choice as scintillator is LuAG:Ce (Ce-doped Lu_3_Al_5_O_12_, Crytur, Czech Republic) due to its excellent light output, radiation hardness and stopping power (Touš *et al.*, 2008 ▸). The thickness of the scintillator is chosen according to the depth of focus of the visible-light scheme, *i.e.* related to numerical aperture and effective pixel size (Koch *et al.*, 1998 ▸). The choice of camera, the desired temporal–spatial resolution and field of view depend on numerous parameters such as the required wavelength, which together with the acquisition rate drives the choice of the insertion device. The latter then defines the maximum field of view.

Since the design of the abuse chamber is focused on *in* *situ* studies, it additionally allows the simultaneous recording of optical images with an optical camera and IR sequences by a thermal imaging camera. This is realized by the two openings installed on the sides of the X-ray windows [see Fig. 2 ▸ (4)]. The adapter mounted onto the openings was designed to allow the camera to be mounted at an angle of 45°, thus ensuring optimal visibility of the cells. If necessary, different adapters can be installed to achieve different viewing angles. The germanium window used has a high-temperature resistance and an optical transmission in the wavelength range 8–12 µm. The IR images were recorded with a VarioCAM head HiRes 640 thermal imaging camera. This camera has a resolution of 640 × 480 pixels and a frame rate of 25 Hz. The resulting recordings can be used in addition to the temperature data from the thermocouples, further serving as a safety measure through contactless monitoring of the chamber interior.

The temperature recording inside the chamber is done with type-K thermocouples, which allow an accurate temperature measurement of up to 1200°C. The thermocouples are attached to the batteries with tape and thermal compound, to ensure an accurate acquisition of the temperature at the battery surface. Additionally, the temperature inside the chamber is also recorded. The voltages of the battery cells are accessed with differential probes and the pressure inside the chamber is recorded with a piezoresistive pressure sensor. All measurement cables can be easily fed through a pre-installed, pressure-proof access port in the wall of the chamber, which moreover adds flexibility for supplementary measurements. This cable setup also facilitates sample exchange between experiments.

The cleaning of the chamber is realized by wiping the surfaces inside the chamber, including the aluminium windows for the X-rays and the germanium window for the IR camera, and removing larger particles with a specialized vacuum cleaner. Preparation includes painting the surface black, insulating the poles with Kapton foil and attaching the measurement leads. After cleaning the chamber, the holder is attached to the sample table of the chamber and the measurement leads connected to the connection box inside the chamber.

In order to compare the data acquisitions across the multiple recording devices, a TransCom CompactX XL2 data acquisition recorder was used. This device enables time-synchronous recording with a high channel density. The wide range of different recording modes (*e.g.* continuous or event-controlled recording) and the various digital in- and output channels enable great flexibility in terms of synchronization at different events during the battery failure. In the current experimental setup, the data were recorded with a sample rate of 1 kHz, but in principle a maximum sample rate of up to 16 MHz per channel is possible.

To facilitate data post-processing, and also allow fast evaluation of the results during the experimental campaign, a data synchronization graphical user interface (GUI) has been developed specifically for the operation of the test environment presented in this paper. This script imports all collected data, including radiography, thermal imaging, temperature/voltage data, and gives the user the ability to easily visualize all measurement parameters synchronized in time. Through visualization, an initial interpretation from the time-synchronized data is thus possible after each experiment. This allows for a progressive adaptation of the measurement matrix within the beam time.

## 4. Application

The movies presented in the supporting information show nail penetration triggered failures (mechanical abuse), imaged with 5000 frames s^−1^ (fps), using an indirect high-speed X-ray detector based on a Photron SA-Z. An intense and partially coherent beam at a photon energy of 85 keV was utilized [wiggler gap of 46 mm, filtered by the aluminium windows of the chamber, and additional copper (1.4 mm), aluminium (3.2 mm) and diamond (1 mm) filters]. The indirect detector used relies on a 1000 µm-thick LuAG:Ce single-crystal scintillator lens coupled to the Photron SA-Z. The camera has a pixel size of 20 µm (1024 × 1024 pixel count), and with the beam height of 12 mm, a 20 mm × 12 mm field of view was obtained by using two Hasselblad lenses in tandem arrangement (100/100, giving a 1× magnification, the camera operated with a region of interest of 1024 × 512 pixels) (Mittone *et al.*, 2017 ▸). A higher magnification (5×) was achieved using a different optical scheme based on Mitutoyo lenses (Optique­Peter, France), a 500 µm-thick LuAG:Ce scintillator and a 0.5 mm copper filter installed 30 cm upstream of the detector for heat-load protection (Douissard *et al.*, 2012 ▸). The radiography was obtained with a beam mean energy of 85 keV, through a wiggler gap of 28 mm.

The nail penetration module was set up for 1 mm intrusion depth, with a speed of 6 mm s^−1^. The nail penetration module consists of a linear actuator with integrated DC motor, a spacer and the nail. This modular design allows adaptation to different experimental setups. The current setup allows speeds up to 8 mm s^−1^ and a maximum compression force of 6 kN. The spacer allows the intrusion depth to be set variably, which means that even multiple cells can be penetrated. The nail penetration module is exchangeable in this chamber, in order to allow penetration in both vertical and horizontal directions. This setup also allows one to investigate the primary (triggered) cell as well as the secondary cell that is the target of propagation.

**Figure 4:**
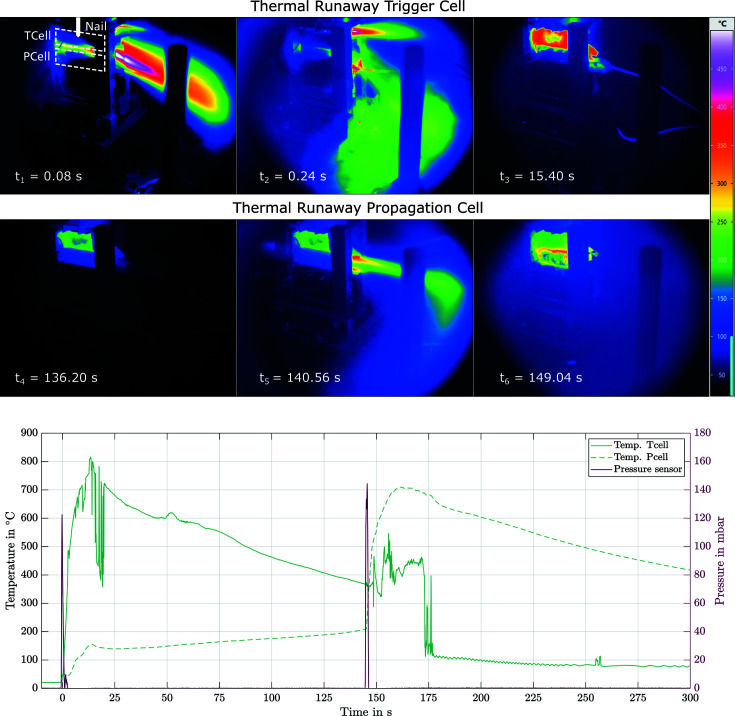
Thermal image of nail-triggered TR of a Molicel P42A cell with another cell mounted below it (Exp.2). The images show the venting process of the first cell and the heat-induced TR of the second cell. The time-synchronous recording of the different data sets enables a comparison with the temperature data of the thermocouple and the pressure differential recorded by the pressure sensor, among other examples.

During the course of the first experiments with the chamber installed at beamline ID19, battery failure propagation was investigated with the two different acquisition setups described above. Two example movies from the experiments are to be found in the supporting information. In the following, we emphasize the possibilities of the presented setup and the resulting insights that can be gained by correlating the different measurement techniques used. For illustration purposes, high-speed X-ray images as well as thermal images for an experiment with an X-ray image area in the trigger cell (Exp.1) and in the propagation cell (Exp.2 and Exp.3) are presented and described below. The cells used are commercially available Molicel INR21700 P42A (Exp.1 and Exp.2) and LG INR21700 M50 (Exp.3). The advantages of IR technology result from the possibility of obtaining a spatially resolved temperature distribution. This is particularly important when the exact position of the decisive temperature change cannot be determined precisely in advance, as is the case, for example, with TR. Furthermore, there is no possibility of losing contact with the cell during the reaction, as can be the case with thermocouples. By recording the measurement data with a common data logger, it is possible to perform a time synchronization at different temporal measurement ranges, recording durations and intervals. This is illustrated in Fig. 4 ▸. These images show a TR caused by the penetration of the first cell with the nail, as well as the steadily increasing temperature on the surface of the second cell and the resulting heat-initiated TR of the second cell. By synchronization, the images of the thermal camera can be compared with the temperature data of the thermocouples and the pressure increase inside the chamber measured with the piezoresistive pressure sensor. Besides the additional information value, the two data sets can also be used to increase accuracy and as a way to calibrate and remove unwanted effects or data artefacts, for example, in the IR measurement technology (reflection, absorption of the germanium window or variations of emissivity due to the melting of the black surface painting). The complete thermal recording of Exp.2 can be found in the supporting information.

**Figure 5:**
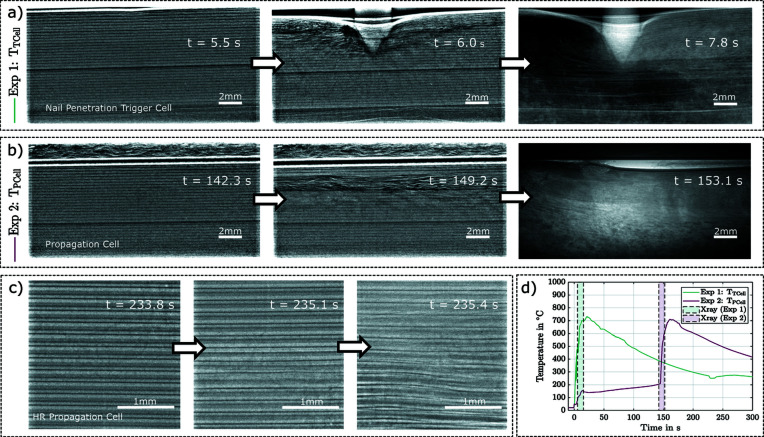
Example images from the high-speed X-ray recording. In Exp.1 (*a*) the cell was punctured directly with the nail, Exp.2 (*b*) and Exp.3 (*c*) show the cells directly under a punctured cell, which also entered a TR due to the temperature rise of the first TR. The setup of the images in (*c*) differs from that of (*a*) and (*b*) by a larger magnification factor (5×), resulting in a pixel size of approximately 4 µm. The images demonstrate the effects of a TR on the internal cell structure. To time the high-speed X-ray images in the abuse process, the images from Exp.1 and Exp.2 are plotted with the temperature data in (*d*). This facilitates the extraction of complex interrelationships across the entire process.

Since the attached sensors and thermal imaging are limited to the cell surface, it is not possible to fully investigate the TR and the processes that mainly take place inside the cell. As mentioned above, high-speed X-ray radiography allows these processes to be investigated. The resulting images enable an examination of the internal structural damage, the propagation of the TR as well as the collapse of the electrolyte layers and the progression of structural decomposition. Fig. 5 ▸ shows examples of images from the high-speed X-ray recordings for experiments Exp.1 (*a*), Exp.2 (*b*) and Exp.3 (*c*). These series of images clearly show the structural changes inside the cell due to the TR. The complete recordings are to be found in the supporting information. These recordings allow a comprehensive and powerful interpretation of the internal processes, with the additional measurement equipment available to monitor the chamber in the relative time sequence of the TR and the processes taking place before and after. Time synchronization allows the time range of the X-ray images to be assigned to the temperature data, as shown in Fig. 5 ▸(*d*). A similar comparison with the thermal images is also possible. This enables extensive investigation of the internal cell processes involved in TR and multi-cell propagation.

## 5. Conclusion

A battery testing chamber is presented that allows lithium-ion battery failures to be investigated at the ID19 X-ray imaging facility. The chamber was designed to withstand the resulting exothermic reaction of a TR including fire, tolerate a significant pressure rise without containment failure, dissipate toxic gases and particles, and additionally provide the ability to suppress a fire by purging with inert gas. The setup allows one to carry out conventional tests with one triggered battery as well as propagation tests with multiple batteries. Furthermore, the setup enables simultaneous observation of the experiment with a variety of complementary *in* *situ* measurement methods. In addition to radiography, temperature and voltage measurements as well as thermal imaging are currently possible, and further extensions to the test setup are planned, including the implementation of a rotation stage to allow for *in* *situ* tomography. A setup-specific software was developed and implemented to allow easy synchronization of the results to facilitate a holistic approach to the analysis of the heterogeneous data. The results demonstrate that the battery testing chamber is capable of combining different measurement methods and therefore allows analysis of processes of different types, timescales and length scales during the abuse process. This will enable a multi-physics approach, which is needed for in-depth understanding of the processes inside a cell during TR. The evaluated data from the first measurement campaign confirm that the testing chamber is capable of providing these meaningful insights into the internal process of TR and multi-cell propagation of current and future energy-storage devices.

## Supplementary Material

M50 propagation cell. DOI: 10.1107/S1600577522010244/ye5024sup1.mp4

P42A propagation. DOI: 10.1107/S1600577522010244/ye5024sup2.mp4

P42A trigger cell. DOI: 10.1107/S1600577522010244/ye5024sup3.mp4

P42A Exp 2. DOI: 10.1107/S1600577522010244/ye5024sup4.mp4
